# An integrated framework for evaluating landscape performance in tourism-oriented rural areas

**DOI:** 10.1038/s41598-025-28784-y

**Published:** 2025-12-08

**Authors:** Yingnan Li, Guangxi Shen, Yuedong Wang, Yakun Chang, Danqing Mo, Peijia Xue, Jingang Li, Lu Ding

**Affiliations:** 1 Department of Environmental Design, Jiangsu University, Zhenjiang, 212013 China; 2 OJEong Resilience Institute, Korea University, Seoul, 02841 Republic of Korea; 3 School of Landscape Architecture, Lincoln University, Christchurch, 7647 New Zealand; 4 School of Architecture and Urban Planning, Jilin Jianzhu University, Changchun, 130118 China; 5 Department of Environmental Science and Ecological Engineering, Korea University, Seoul, 02841 Republic of Korea; 6 Department of Computer Graphics Technology, Purdue University, West Lafayette, 47907 USA; 7 Environmental Planning Institute, Seoul National University, Seoul, 08826 Republic of Korea

**Keywords:** Landscape performance evaluation, Rural tourism, Sustainable development, Analytic hierarchy process, Rural revitalization, Environmental economics, Sustainability

## Abstract

Rural tourism has emerged as a key strategy for regional revitalization and sustainable development, yet the lack of a comprehensive and context-sensitive framework to evaluate landscape performance remains a critical gap. In this study, we developed a landscape performance evaluation (LPE) framework tailored to tourism-oriented rural areas by integrating the analytic hierarchy process with multidimensional indicators. Using Jiangxin Island as a case study, the framework was used to assess landscape sustainability across environmental, economic, social, and aesthetic dimensions. The results show that the overall landscape performance score for Jiangxin Island is 3.66, placing it in the Good category. Among the four major dimensions, environmental performance holds the highest weight (0.34) and scores 3.80, reflecting effective ecological protection and water quality management, while also revealing challenges in flood control and biodiversity conservation. Economic performance (weight: 0.24) achieves the highest score of 4.95, driven by increased employment and tourism revenue, though income sources remain concentrated in traditional sectors such as agriculture and hospitality. In contrast, social performance, despite its relatively high weight (0.28), scores the lowest at 2.72, indicating deficiencies in infrastructure, accessibility, and cultural experience. Aesthetic performance, with the lowest weight (0.14) and a score of 3.00, highlights generally acceptable visual conditions while still indicating the need for improved overall landscape coherence. These differentiated results not only validate the robustness of the proposed framework but also provide practical guidance for rural tourism development. Based on the findings, targeted strategies are recommended—such as enhancing infrastructure, improving biodiversity management, diversifying economic activities, and strengthening local cultural identity. This study contributes to the theoretical and practical understanding of landscape performance in tourism-oriented rural areas and offers a replicable framework to inform sustainable agricultural land management and multifunctional landscape planning in broader rural development contexts.

**Supplementary Information:**

The online version contains supplementary material available at 10.1038/s41598-025-28784-y.

## Introduction

In the past decades, rural tourism has emerged as a critical approach to sustainable development^1^. According to the European Landscape Convention^2^, landscape is defined as “an area, as perceived by people, whose character is the result of the action and interaction of natural and/or human factors.” This perspective emphasizes both the physical and perceptual dimensions of landscape, making it particularly relevant in the context of tourism. In rural development, landscapes not only support ecological restoration and cultural heritage preservation^3,4^ but also provide critical assets for tourism by offering aesthetic experiences, delivering ecosystem services, and conveying socio-cultural values^5,6^. With increasing global attention to environmental sustainability, there is growing demand for comprehensive assessment approaches that can enhance visitor satisfaction and support the balanced development of environmental, social, and economic functions^7,8^. This demand has prompted researchers to explore integrated performance evaluation frameworks to support long-term rural tourism development.

In recent years, landscape performance evaluation (LPE) has emerged as a significant topic in landscape research and planning. Landscape performance refers to the measurable benefits that a landscape provides in terms of ecological function, social well-being, economic value, and visual experience^9^. Its objective is to assess the multiple benefits of landscapes, including environmental, social, economic, and increasingly aesthetic dimensions, through both quantitative and qualitative methods, thereby supporting relatively more informed landscape design and management decisions^10^. The theoretical framework of LPE originated from the assessment of ecosystem services and has gradually expanded to include social well-being, cultural values, and visual perception, forming a relatively more comprehensive and multidimensional evaluation approach^11^. Existing frameworks, such as the Landscape Performance Series (LPS) introduced by the Landscape Architecture Foundation (LAF), have substantially contributed to urban park and green infrastructure assessments, emphasizing ecological and economic outputs^12,13^. However, they offer limited consideration of place-specific aesthetic, socio-cultural, and tourism-related values, especially in rural contexts. On the contrary, tourism-oriented rural landscapes serve ecological, economic, and cultural functions simultaneously, requiring a relatively more integrated and context-sensitive evaluation framework^14^. Despite the increasing interest in LPE applications, research in such rural settings remains limited. Most existing studies prioritize ecological performance while overlooking perceptual and economic dimensions and rely heavily on single-layered indicators that fail to capture the complexity and dynamism of tourism-based rural systems^15,16^. Additionally, as highlighted by Lane^17^, who emphasizes the importance of holistic approaches in rural development, such a framework can enhance the sustainability of rural tourism destinations and offer scientific support for policy formulation and evidence-based planning.

Previous research has explored the multifaceted value of rural landscapes in ecosystem services, cultural preservation, and socio-economic functions^18^. For example, according to the study by Chen et al.^19^, rural landscapes play a crucial role in balancing agricultural development and biodiversity conservation. Similarly, Bohnet et al.^20^ demonstrated that rural landscapes hold ecological, cultural, social, and economic value, functioning as spaces for heritage preservation, biodiversity conservation, community interaction, and sustainable development. However, previous research did not account for the interactions between landscape aesthetics, cultural identity, and local economic vitality. Several studies have sought to bridge this gap. For example, Howley^21^ conducted a comprehensive empirical study assessing public preferences for rural landscape aesthetics, demonstrating that attributes such as greenery, openness, and natural features significantly influence visual appreciation and landscape evaluation. In a related study, Carneiro et al.^22^ reported that both visual features, such as vegetation and built heritage, and non-visual elements, e.g., sounds, smells, and social interactions, significantly shape tourists’ perceptions of rural landscapes. Their findings underscore the multisensory and experiential nature of landscape evaluation in tourism contexts^22^. Traditional assessment methodologies are often constrained by static ecological indicators, making it difficult to capture the evolving nature of tourism-oriented rural landscapes, particularly in terms of visitor experiences, participatory cultural engagement, and adaptive landscape management^23^. Therefore, there is a pressing need for a more adaptable LPE framework for tourism-oriented rural areas, which can integrate diverse factors and provide actionable guidance for rural tourism planning and management. To address this gap, in this study, we adopted the analytic hierarchy process (AHP), a structured multi-criteria decision-making method widely applied in landscape and environmental planning. AHP enables the systematic weighting of diverse indicators through pairwise comparisons, ensuring transparency, consistency, and the integration of expert judgment^24^. Its flexibility and clarity make it particularly suitable for evaluating the complex and multidimensional characteristics of rural tourism landscapes.

Although rural tourism development has adopted diverse forms globally, China presents a particularly illustrative case owing to the integration of tourism within its broader national strategies for rural revitalization. In recent years, rural tourism has undergone rapid development in China, becoming a critical part of the rural revitalization strategy^25^. Government policies, such as the National Rural Revitalization Strategy Plan (2018–2022), have emphasized the role of tourism in driving rural economic growth, preserving cultural heritage, and advancing sustainable development^26,27^. Internationally, rural tourism is also increasingly framed as an integrative strategy, linking economic revitalization, landscape value, and community participation, as seen in examples from Portugal and other regions^22,28^. Rural tourism not only boosts the rural economy but also fosters urban–rural integration by promoting agricultural sales, driving the growth of the service industry, and creating employment opportunities for rural residents^29^. It also enhances cultural preservation through interactions between tourists and local communities^30^. Moreover, the development of ecotourism and leisure agriculture helps reduce environmental damage from traditional agriculture while promoting ecological restoration^31,32^. Despite these achievements, challenges such as uneven landscape quality, overdevelopment of tourism resources, and inadequate management remain. These challenges indicate the need for a scientific evaluation system to guide the sustainable development of rural tourism.

Rural tourism has been increasingly recognized as a catalyst for sustainable regional development; however, existing LPE frameworks often focus on urban or ecological restoration contexts, lacking tailored approaches for tourism-driven rural areas. Additionally, structured decision-making methods such as AHP are rarely integrated into rural LPE, resulting in limited methodological rigor and multidimensional assessment. To address these gaps, this study aimed to establish an LPE framework applicable to tourism-oriented rural areas for assessing the comprehensive benefits of rural landscapes in tourism development. We further demonstrated the practical applicability of the framework by conducting an empirical study on the tourism-oriented rural area of Jiangxin Island in Zhenjiang, China as a case and offer theoretical and methodological support for optimizing rural landscape planning and promoting sustainable tourism development. Accordingly, we mainly addressed the following research questions: 1) What are the key components and structure of a comprehensive LPE framework suitable for tourism-oriented rural areas, integrating environmental, social, economic, and aesthetic dimensions? 2) How can the relative importance (weights) of indicators within each dimension be systematically determined using AHP? 3) To what extent can the proposed LPE framework be effectively applied and validated through a case study of Jiangxin Island, and what improvement strategies can be derived based on the evaluation results? The findings of this study offer a scientifically grounded approach for assessing and enhancing the sustainability of rural tourism landscapes by answering these questions.

## Method

### Study site

**Fig. 1:**
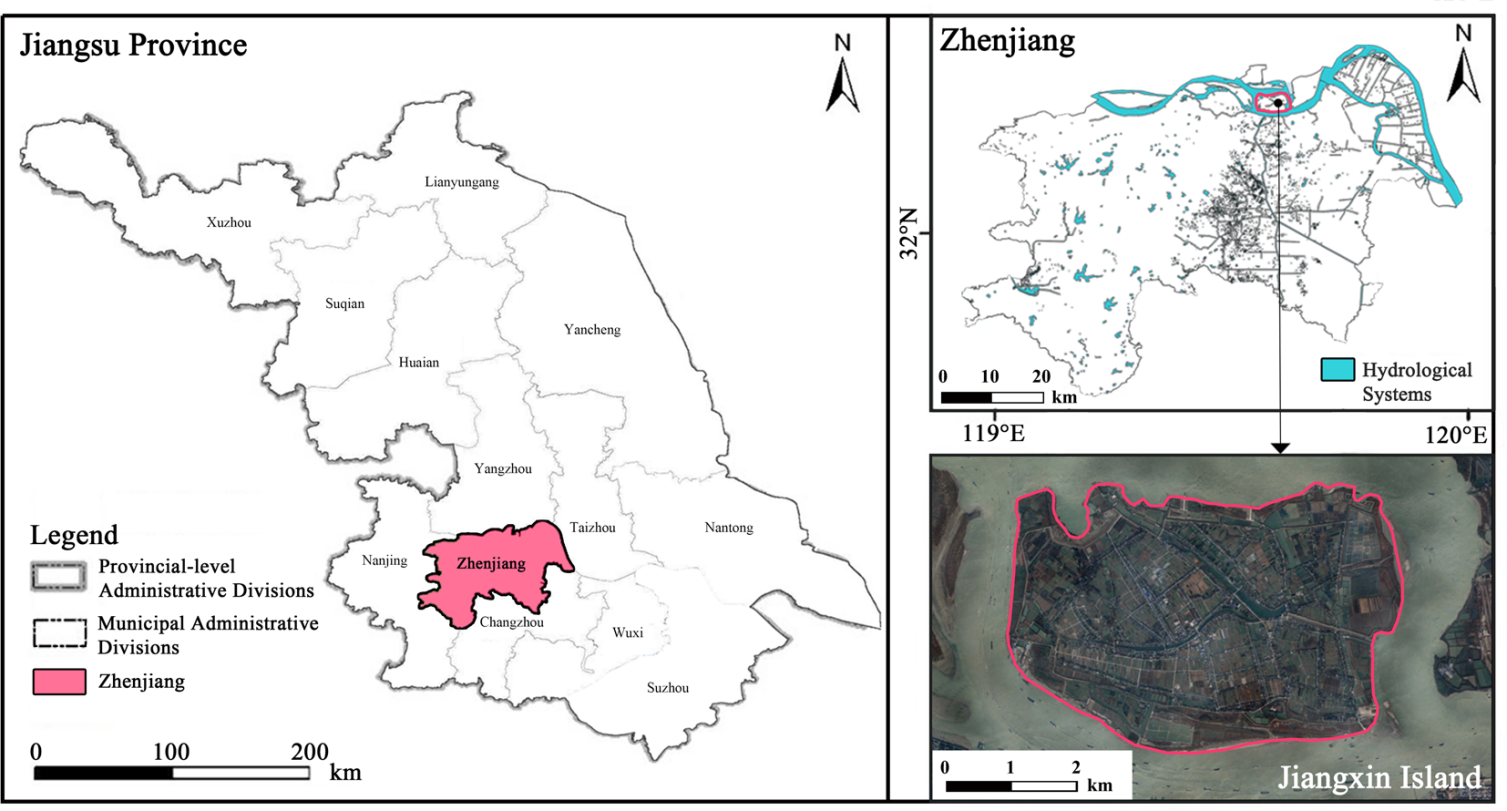
Location of study site. (The map was created using the open-source GIS software QGIS (version 3.40). The base data for the administrative divisions and hydrological systems was obtained from OpenStreetMap. The satellite imagery of Jiangxin Island was acquired from Amap.)

Jiangxin Island is located in Zhenjiang City, China (119°E, 31°N) (Fig. 1), which has a humid subtropical climate with four distinct seasons, featuring hot and humid summers and mild winters^33,34^. The island itself is a sandbank formed by sediment deposition from the Yangtze River, covering an area of 13.46 km^2^. It has predominantly flat terrain and is enclosed by river embankments along the Yangtze River, with agriculture and tourism serving as its primary industries. The island is characterized by high vegetation coverage and a well-developed hydrological network, including numerous rivers, ponds, and paddy fields.

Owing to its geographical isolation from the mainland, Jiangxin Island remains relatively insulated from urban pollutants, preserving its natural environment and fostering a favorable microclimate. To protect endangered species, such as the Yangtze finless porpoise (*Neophocaena asiaeorientalis*), a designated conservation area has been established on the northern side of the island. This initiative has led to stringent restrictions on industrial activities along the shoreline, contributing to the maintenance of high levels of water quality. The protected area is characterized by extensive wetlands and abundant fish resources, playing a vital role in sustaining the island’s ecological balance.

Using the national standard "Classification, Investigation, and Evaluation of Tourism Resources," implemented on July 1, 2018, we evaluated the tourism resources of Jiangxin Island, Zhenjiang. According to this standard, the tourism resources of Jiangxin Island can be categorized into 6 main categories and 10 subcategories (Appendix Table A1).

**Table 1:** LPE framework for the tourism-oriented rural area of Jiangxin Island.

Goal layer	Criteria layer	Indicator layer
LPE framework for tourism-oriented rural area of Jiangxin Island	Environmental performance (B1)	Water quality status (C1)
		Flood control (C2)
		Habitat protection (C3)
		Plant diversity (C4)
		Green space ratio (C5)
		Air quality (C6)
	Social performance (B2)	Leisure and entertainment (C7)
		Science education (C8)
		Infrastructure completeness (C9)
		Convenience of tourist routes (C10)
		Enhancement of urban image (C11)
	Economic performance (B3)	Job creation (C12)
		Tourism consumption (C13)
	Aesthetic performance (B4)	Residential architectural features (C14)
		Landscape quality (C15)
		Landscape harmony (C16)

### Establishing an LPE framework for tourism-oriented rural landscapes

The LAF in the United States introduced LPS in 2010, focusing on three key dimensions: economic, social, and environmental performance^35^. LPS offers a flexible framework for evaluating landscape performance, which is organized into 7 major categories and further subdivided into 36 subcategories. Additionally, LPS has established a dedicated platform for sharing landscape performance research, where data collection methods, sources, calculation processes, and project outcomes are presented through case study briefs. To date, the platform has documented 155 case studies covering 30 different landscape types (https://www.landscapeperformance.org/).

The LPS framework, centered on economic, social, and environmental factors, provides a universal foundation for LPE^36^. Considering its alignment with the objectives of performance assessment in tourism-oriented rural landscapes, this study adopts LPS evaluation indicators as a reference, with necessary modifications tailored to the specific characteristics of Jiangxin Island.

Building upon the structure provided by LPS, we integrated the developmental objectives and characteristics of tourism-oriented rural landscapes while drawing insights from existing literature on rural landscapes and tourism indicator systems. Through the integration of expert judgment (subjective weighting via AHP) and quantitative data analysis (objective evaluation via composite index method), an LPE framework was developed for tourism-oriented rural area of Jiangxin Island. This framework is structured into 4 primary performance dimensions—environmental, social, economic, and aesthetic—and further subdivided into 16 evaluation indicators (Table 1), which were selected based on their theoretical significance and contextual relevance. Specifically, environmental indicators such as water quality, flood control, and plant diversity reflect the island’s role as an ecological conservation zone and drinking water source. Social indicators such as infrastructure completeness, tourist route convenience, and educational value align with tourism planning priorities. Economic indicators such as job creation and tourism consumption capture tourism-driven development outcomes, while aesthetic indicators such as residential architectural features and landscape harmony emphasize the visual integration of natural and rural elements. Together, these indicators provide a comprehensive basis for assessing the multifunctional landscape performance of Jiangxin Island in the context of sustainable rural tourism.

### Determining the weights of LPE indicators for tourism-oriented rural areas

To determine the weights of evaluation indicators, we applied the AHP method. A questionnaire survey (Table A2) was conducted among experts in relevant fields. The collected data were statistically analyzed to derive the final weight values. This process established the relative importance of each hierarchical level and its corresponding indicators, ensuring a systematic and objective assessment of the landscape performance for tourism-oriented rural area of Jiangxin Island.

#### Steps for determining weights of the criteria level

*Step* 1: construction of judgment matrices.

Judgment matrices were constructed for both the criteria and indicator levels in the Jiangxin Island LPE framework. A 1–5 scale was used for the pairwise comparisons of indicators, with experts following the detailed guidelines provided in the scoring forms to ensure consistency in their evaluations.

Twelve experts were selected based on their academic and professional experience across landscape architecture, rural planning, environmental management, and tourism development, ensuring interdisciplinary perspectives and capturing diverse rural contexts within eastern China. Their responses were averaged to generate the judgment matrix. The analysis was performed using the SPSSAU statistical platform.

*Step* 2: construction of the criteria level judgment matrix.

The criteria level includes four core dimensions: environmental, social, economic, and aesthetic performance. In this step, a pairwise comparison matrix was developed to determine the relative importance of these dimensions, based on expert assessments following the AHP method. The experts compared each dimension in terms of its contribution to overall landscape performance in tourism-oriented rural areas. The aggregated judgment matrix is shown in Table 2.

**Table 2:** Judgment matrix for the criteria layer.

Criteria layer	Environmental performance (B1)	Social performance (B2)	Economic performance (B3)	Aesthetic performance (B4)
Environmental performance (B1)	1	1	2	2
Social performance (B2)	1	1	1	2
Economic performance (B3)	1/2	1	1	2
Aesthetic performance (B4)	1/2	1/2	1/2	1

*Step* 3: AHP-based analysis and weight calculation.

An AHP-based hierarchical analysis was performed using the sum–product method, and the analysis results are shown in Table 3. The eigenvector and corresponding weights were computed, with the environmental, social, economic, and aesthetic performances assigned the weight values of 33.73%, 28.18%, 24.01%, and 14.09%, respectively. The maximum eigenvalue (λ_max_) was calculated as 4.06, and the consistency index (CI) was computed as 0.02.

**Table 3:** Results of the AHP-based hierarchical analysis.

AHP-based hierarchical analysis results				
Criteria layer	Eigenvector	Weight (%)	Maximum Eigenvalue (λmax)	CI
Environmental performance (B1)	1.35	33.73%	4.06	0.02
Social performance (B2)	1.13	28.18%		
Economic performance (B3)	0.96	24.01%		
Aesthetic performance (B4)	0.56	14.09%		

*Step* 4: consistency testing

To verify the consistency of the judgment matrix, the consistency index (CI) was calculated using the formula: CI = ( λ_max_​− n)/(n − 1), and the consistency ratio (CR) was then obtained as CR = CI/RI, where the random index (RI) = 0.89 (obtained from the RI table for a 4 × 4 matrix). The computed CR value was 0.02, which is less than 0.10, indicating that the matrix passes the consistency test^37^.

#### Steps for determining weights of indicator layer

Table 4 presents the judgment matrix for the environmental performance indicators, constructed based on expert evaluations. A total of 12 experts specializing in relevant fields participated in scoring the environmental performance indicator layer. Their individual scores were averaged to generate the final judgment matrix (Table 4). The AHP-based analysis was performed using the SPSSAU platform to derive the weight values of the environmental performance indicators.

**Table 4:** Judgment matrix for the environmental performance.

Environmental performance indicators (B1)	C1	C2	C3	C4	C5	C6
Water quality (C1)	1	2	1/2	1	2	1
Flood control (C2)	1/2	1	1/2	1	1	1
Habitat protection (C3)	2	2	1	2	1/2	1
Plant diversity (C4)	1	1	1	1	1	1
Green space ratio (C5)	1/2	1	2	1	1	2
Air quality (C6)	1	1	1	1	1/2	1

Based on the judgment matrix shown in Table 5, a 6 × 6 judgment matrix was constructed to evaluate 6 aspects: water quality, flood control, habitat protection, plant diversity, green space ratio, and air quality. Using AHP with the sum–product method, the analysis yielded the eigenvector values for these 6 aspects (1.14, 0.77, 1.14, 0.94, 1.16, and 0.85, respectively), which were used to determine their corresponding weights. Specifically, weights of 19.05%, 12.80%, 19.05%, 15.58%, 19.35%, and 14.19% were assigned to water quality, flood control, habitat protection, plant diversity, green space ratio, and air quality, respectively.

**Table 5:** Results of the environmental performance weights.

AHP-based hierarchical analysis results				
Indicators	Eigenvector	Weight (%)	Maximum Eigenvalue (λmax)	CI
Water quality (C1)	1.14	19.05%	6.39	0.08
Flood control (C2)	0.77	12.80%		
Habitat protection (C3)	1.14	19.05%		
Plant diversity (C4)	0.94	15.58%		
Green space ratio (C5)	1.16	19.35%		
Air quality (C6)	0.85	14.19%		

To verify the consistency of the judgment matrix, the maximum eigenvalue was calculated as 6.39, and the CI was derived as 0.08. Based on the RI table, the RI value for a 6 × 6 judgment matrix was determined to be 1.26, resulting in a CR value of 0.06. As the CR value is less than 0.10, the judgment matrix demonstrated satisfactory consistency, confirming the logical validity of the expert evaluations. Accordingly, this weight distribution framework serves as a key basis for environmental performance assessment, providing essential data support for further comprehensive analysis.

The analysis of the social performance matrix (Table 6) using AHP yielded the following weight values: 28.08%, 25.35%, 19.61%, 19.09%, and 7.88% for leisure and entertainment value, science education, infrastructure completeness, convenience of tourist routes, and enhancement of urban image, respectively (Table 7). The consistency test confirmed the reliability of these weights, with a maximum eigenvalue of 5.42, a CI of 0.10, and a CR of 0.09, all within acceptable limits. These results provide a structured basis for assessing social performance in tourism-oriented rural landscapes.

**Table 6:** Judgment matrix for the social performance.

Social Performance (B2)	C7	C8	C9	C10	C11
Leisure and entertainment (C7)	1	2	2	1	2
Science education (C8)	1/2	1	2	2	3
Infrastructure completeness (C9)	1/2	1/2	1	2	3
Convenience of tourist routes (C10)	1	1/2	1/2	1	4
Enhancement of urban image (C11)	1/2	1/3	1/3	1/4	1

**Table 7:** Results of the social performance weights.

AHP-based hierarchical analysis results				
Indicators	Eigenvector	Weight (%)	Maximum Eigenvalue (λmax)	CI
Leisure and entertainment (C7)	1.40	28.08%	5.42	0.10
Science education (C8)	1.27	25.35%		
Infrastructure completeness (C9)	0.98	19.61%		
Convenience of tourist routes (C10)	0.96	19.09%		
Enhancement of urban image (C11)	0.39	7.88%		

The economic performance analysis (Table 8) evaluated 2 key indicators: job creation and tourism consumption. Based on expert assessments, the AHP-based analysis determined that tourism consumption holds greater weight in economic performance compared to job creation. The judgment matrix was processed using SPSSAU, and the resulting weights and consistency test results are presented in Table 9.

**Table 8:** Judgment matrix for the economic performance.

Economic Performance (B3)	C12	C13
Job creation (C12)	1	1/3
Tourism consumption (C13)	3	1

**Table 9:** Results of the economic performance weights.

AHP-based hierarchical analysis results				
Indicators	Eigenvector	Weight (%)	Maximum Eigenvalue (λmax)	CI
Job creation (C12)	0.50	25%	2	0
Tourism consumption (C13)	1.50	75%		

Based on the expert scores, a 2 × 2 judgment matrix was constructed, and an AHP-based analysis was performed using the geometric mean method. The eigenvector components for job creation and tourism consumption were 0.50 and 1.50, corresponding to weights of 25% and 75%, respectively (Table 9). The maximum eigenvalue was calculated as 2, resulting in a CI value of 0. As the RI for a 2 × 2 matrix is 0 and CI is always 0, a consistency test is not required.

**Table 10:** Judgment matrix of the aesthetic performance.

Aesthetic Performance (B4)	C14	C15	C16
Residential architectural features (C14)	1	1	2
Landscape quality (C15)	1	1	2
Landscape harmony (C16)	1/2	1/2	1

Table 10 presents the judgment matrix for the aesthetic performance, where 12 experts evaluated the corresponding indicators. Their individual scores were averaged to obtain the final judgment matrix. The AHP-based analysis was performed using the SPSSAU statistical platform, and the results are shown in Table 11.

**Table 11:** Results of the aesthetic performance weights.

AHP-based hierarchical analysis results				
Indicators	Eigenvector	Weight (%)	Maximum Eigenvalue (λmax)	CI
Residential architectural features (C14)	1.20	40%	3	0
Landscape quality (C15)	1.20	40%		
Landscape harmony (C16)	0.60	20%		

Based on the data, a 3 × 3 judgment matrix was constructed to assess 3 aspects: residential architectural features, landscape quality, and landscape harmony. Using AHP with the geometric mean method, the analysis yielded eigenvector components of 1.20, 1.20, and 0.60 for the 3 indicators, corresponding to weights of 40%, 40%, and 20%, respectively. The maximum eigenvalue was 3, resulting in a CI value of 0. Given that the RI for a 3 × 3 matrix is 0.52, the CR was calculated as 0, indicating that the matrix satisfies the consistency requirement.

The smaller the CR value, the better the consistency of the judgment matrix. Generally, when CR is below 0.1, the matrix passes the consistency test. However, when CR exceeds 0.1, the matrix is considered inconsistency and must be adjusted before reanalysis. The RI values for different matrix orders are presented in Table 12.

**Table 12:** RI values.

RI values														
n-order	3	4	5	6	7	8	9	10	11	12	13	14	15	16
RI value	0.52	0.89	1.12	1.26	1.36	1.41	1.46	1.49	1.52	1.54	1.56	1.58	1.59	1.59

#### Comprehensive ranking and consistency check

1. Calculation of overall weights.

In the hierarchical structure at level h, where $C_{h}$ is the lowest level of the hierarchy and $C_{1}$ is the highest level, $W_{i}$ represents the relative weight of the lower-level indicators to the upper-level indicators, and $C_{h}$‘s overall weight is $W_{z}$.

The weight vector is represented as:

1 $$W = w_{h} w_{h - 1} w_{h - 2} \ldots w_{1}$$

(2) Overall consistency check.

The one-time consistency check for the overall ranking is calculated using:

2 $$CR = \frac{{a_{1} CI_{1} + a_{2} CI_{2} + \ldots + a_{m} CI_{m} }}{{a_{1} RI_{1} + a_{2} RI_{2} + \ldots + a_{m} RI_{m} }}{ }$$

When $CR$ < 0.1, the overall ranking passes the consistency check.

### Constructing an evaluation model using the comprehensive index method

After determining the indicator weights at each level, the comprehensive evaluation index for the landscape performance of the tourism-oriented rural area of Jiangxin Island was calculated by aggregating individual indicator scores. This index provides a holistic evaluation of the project’s landscape performance outcomes.

Tourism-oriented rural areas face distinct environmental challenges compared to general tourism destinations. Their landscape systems are often intertwined with social issues, such as improving farmers’ income and supporting rural economic development^38^. As a result, these areas require an evaluation framework that accounts for multiple influencing factors^39.^ Therefore, the landscape evaluation framework for Jiangxin Island must provide multidimensional feedback on both current conditions and future development trends, necessitating a comprehensive and systematic evaluation model.

The comprehensive index method effectively addresses this challenge by enabling an integrated evaluation that incorporates multiple influencing factors. Before formal calculation, the positive and negative indicators were adjusted to ensure consistency, with all indicators converted into positive direction.

The comprehensive evaluation index is calculated using the following formula:

3 $$BCD = \sum W_{i} D_{i}$$

where BCD represents the landscape performance index of the tourism-oriented rural areas, Wi denotes the weight of each indicator, and Di is the evaluation score of each indicator.

The overall landscape performance score for the tourism-oriented rural area in Jiangxin Island was determined using the comprehensive index method, providing insights into its current status and future potential. A dimensionless transformation was applied to normalize the landscape performance scores. Subsequently, based on the total evaluation score derived from indicator weights, the results were classified into five levels: Excellent, Good, Average, Poor, and Very Poor, as detailed in Table 13.

**Table 13:** Evaluation criteria for the tourism-oriented rural area of Jiangxin Island.

Evaluation level	Score	Description of the evaluation level
Very poor	0 ≤ Score < 1	The study area exhibits severe environmental, economic, social, and aesthetic issues, making it unsustainable. There is a high risk of failure, and immediate corrective actions are necessary.
Poor	1 ≤ Score < 2	The study area has noticeable weaknesses in environmental protection, economic contribution, social benefits, and landscape aesthetics. Visitor dissatisfaction may potentially reduce tourism appeal, indicating that targeted improvements are needed.
Average	2 ≤ Score < 3	The study area performs moderately across various aspects, with potential for improvement in ecological conservation and visitor satisfaction. It contributes to local economic and social development, but specific weaknesses still need to be addressed.
Good	3 ≤ Score < 4	The study area performs well in environmental, economic, social, and aesthetic dimensions. Ecological protection is effective, and tourism positively contributes to the local economy. Visitors express satisfaction, and the landscape quality is commendable. Efforts should focus on maintaining and further enhancing these strengths.
Excellent	4 ≤ Score ≤ 5	The study area excels in all aspects, demonstrating sustainable development and high visitor satisfaction. Tourism is thriving, supported by well-developed infrastructure. This standard should be maintained, and further efforts should be made to position the project as a flagship attraction within Zhenjiang’s tourism sector.

Subjective indicators, such as aesthetic quality and cultural perceptions, were quantified using Likert-scale responses (1–5) collected from visitor surveys and expert assessments, and the resulting scores were normalized for consistency. These scores were then weighted using the AHP-derived weights and aggregated through the comprehensive index method to generate the final dimension-level scores and the overall performance score.

### On-site survey and data collection

The on-site data mainly consisted of two parts .

(1) Collection of basic data, which was obtained from publicly available information from the Jiangxin Island Ecological Agriculture Park, statistical data from the Zhenjiang Dantu District Statistics Bureau, data from the Zhenjiang Transportation Bureau, and remote sensing data interpretation from Sentinel-2.

(2) On-site survey data, which was collected through on-site photography, on-site sampling and measurement, surveys based on the landscape performance evaluation indicators for tourism-oriented rural area of Jiangxin Island, as well as interviews and questionnaires distributed to tourists, residents, staff, and researchers to obtain relevant indicator data. The questionnaire items were evaluated using a five-point response scale (Very Dissatisfied, Dissatisfied, Neutral, Satisfied, Very Satisfied), as shown in Appendix Table A3.

To minimize seasonal effects, the surveys were conducted during two periods: July 2022 and October 2022. A total of 37 questionnaires were distributed in July, and 63 questionnaires were distributed in October. The determination of plant diversity-related indicators was conducted through on-site measurements using a random sampling method. A total of 10 sample plots were established, each measuring 10 m × 10 m. All field investigations were carried out following the i-Tree Eco v6 guidelines. Trees with a diameter at breast height (DBH) of 4.5 cm or greater and a height of more than 1.3 m were included in this survey.

The instruments used for measurement included an optical rangefinder and a 30-m-long tape measure. The collected plant information included: (1) species, (2) site type, (3) DBH, (4) tree height, (5) crown size, (6) crown loss, (7) mortality rate, and (8) crown exposure. These data were recorded in an Excel spreadsheet to assess the related diversity and structure.

(Three statements for ethics:

1. This study was performed in accordance with Declaration of Helsinki.
2. This study was approved by Jiangsu University.
3. The informed consent was obtained from all participants involved in this study.)

## Results

### Weight calculation results

Based on the pairwise comparison matrices and consistency testing results, the final weights of the landscape performance indicators were determined at all hierarchical levels (Table 14). Among the four primary dimensions, environmental performance received the highest weight (0.34), indicating its central role in the evaluation framework. It was followed by social performance (0.28), economic performance (0.24), and aesthetic performance (0.14). At the sub-indicator level, tourism consumption (C13) was assigned the highest overall weight (0.18), highlighting its substantial impact on the perceived success of rural tourism landscapes. Other influential indicators included leisure and entertainment value (C7), green space ratio (C5), and habitat protection (C3), all of which reflect the importance of both ecological quality and recreational experience in shaping overall landscape performance. The results revealed a strong prioritization of environmental and experiential aspects in the expert evaluation process, aligning with sustainability-oriented planning goals. The weight system also provided a clear structure for interpreting evaluation results in subsequent sections, offering practical implications for resource allocation and management focus in a similar rural tourism context.

**Table 14:** Comprehensive weights of the LPE for tourism-oriented rural area of Jiangxin Island.

Goal layer	Criteria layer	Weight	Indicator layer	Weight	Comprehensive weight
LPE framework for tourism-oriented rural area of Jiangxin Island	Environmental performance (B1)	0.34	Water quality (C1)	0.19	0.06
			Flood control (C2)	0.13	0.04
			Habitat protection (C3)	0.19	0.06
			Plant diversity (C4)	0.16	0.05
			Green space ratio (C5)	0.19	0.07
			Air quality (C6)	0.14	0.05
	Social performance (B2)	0.28	Leisure and entertainment (C7)	0.28	0.08
			Science education (C8)	0.25	0.07
			Infrastructure completeness (C9)	0.20	0.06
			Convenience of tourist routes (C10)	0.19	0.05
			Enhancement of urban image (C11)	0.08	0.02
	Economic performance (B3)	0.24	Job creation (C12)	0.25	0.06
			Tourism consumption (C13)	0.75	0.18
	Aesthetic performance (B4)	0.14	Residential architectural features (C14)	0.40	0.06
			Landscape quality (C15)	0.40	0.06
			Landscape harmony (C16)	0.20	0.03

### Basic information statistics of the survey questionnaire

**Fig. 2:**
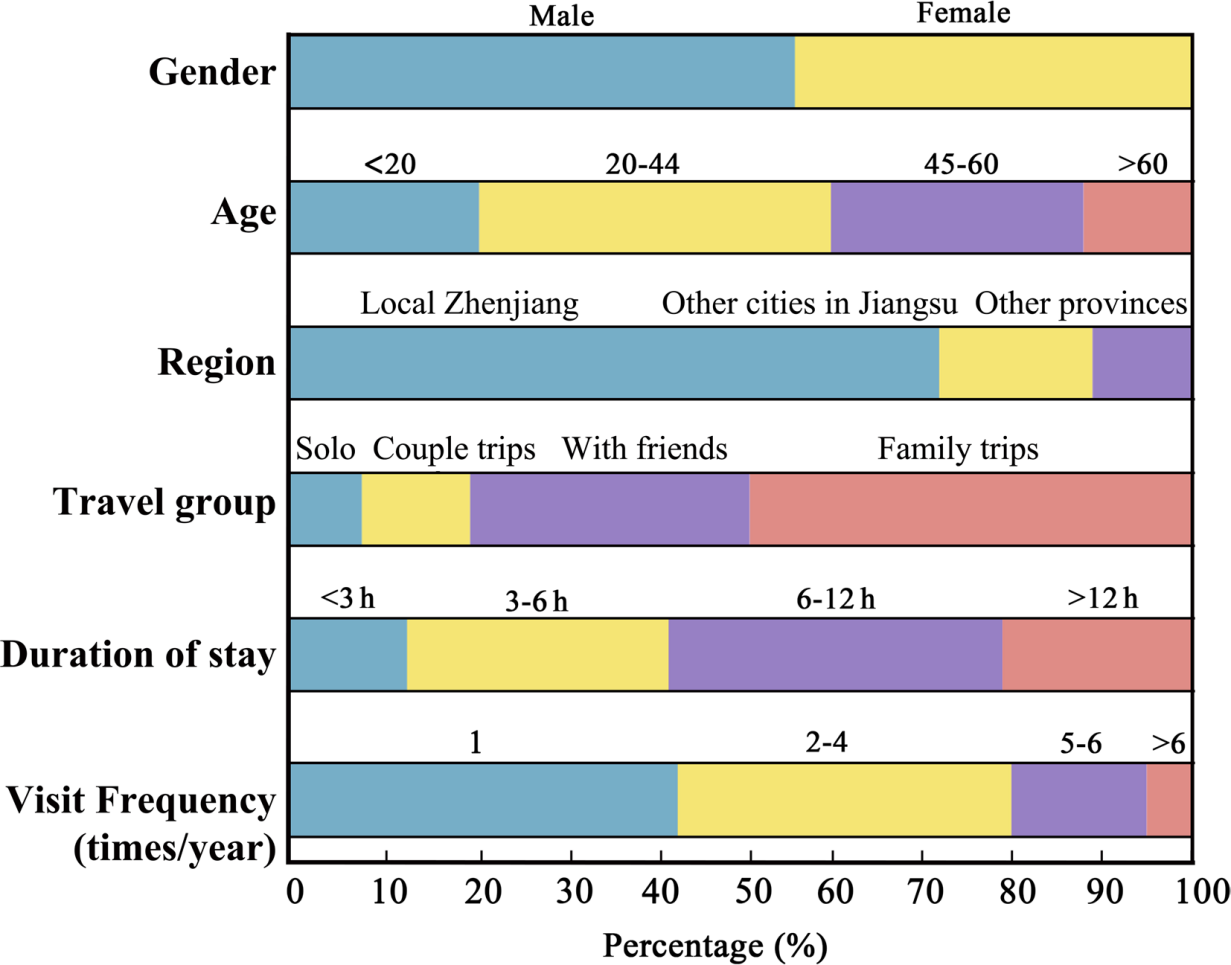
Visitor demographics and travel patterns of survey respondents.

A total of 100 valid questionnaires regarding visitor demographics and travel patterns were collected for this study (Fig. 2). The gender distribution was relatively balanced, with 56% male and 44% female respondents. In terms of age, the majority of visitors (39%) were between 20–44 years old, followed by those aged 45–60 (28%), under 20 (21%), and over 60 (12%), indicating that young and middle-aged adults constitute the core visitor demographic, likely because of their greater mobility and interest in tourism activities. Regarding place of origin, 72% of respondents were from Zhenjiang, reflecting strong local engagement, whereas 17% came from other cities in Jiangsu Province and 11% from outside the province, suggesting broader regional appeal. Travel group composition further revealed that family trips were the dominant choice (49%), followed by trips with friends (31%), couple trips (12%), and solo travel (8%), highlighting the family-friendly character of the destination and its capacity to cater to diverse visitor groups.

Additionally, duration of stay and visit frequency offer further insights into tourist behavior. The most common length of stay was 6–12 h (37%), followed by 3–6 h (29%), more than 12 h (21%), and less than 3 h (13%), which suggests that the island primarily functions as a day-trip destination, though a notable share of visitors stay longer, likely because of accommodation options or immersive activities. In terms of visit frequency, 43% of respondents reported visiting once a year, and 37% visited 2–4 times annually, indicating that most visitors consider the site an occasional leisure destination. Moreover, 15% and 5% visited 5–6 times and more than 6 times annually, respectively, and these frequent visitors largely comprised local residents and commuting workers. This pattern illustrates the dual function of Jiangxin Island as both a recreational space and a daily-use area for the local population. Together, these demographic and behavioral patterns provided foundational context for interpreting subsequent performance evaluations, particularly in terms of social and aesthetic dimensions.

### Results of the LPE for Jiangxin Island

#### Environmental performance evaluation

The environmental performance evaluation of Jiangxin Island encompassed key aspects, such as water quality, flood control, habitat protection, plant diversity, and green space coverage. As one of the five centralized drinking water sources for Zhenjiang, the water quality of the island is of significant concern. According to the water quality data of Zhenjiang’s Centralized Drinking Water Sources (June 2021), assessments based on the Surface Water Environmental Quality Standards (GB3838-2002) and a single-factor evaluation method revealed that the island’s intake from the Yangtze River met the Grade II standard for 59 of the 62 monitored indicators. All of these indicators achieved compliance, resulting in a 100% compliance rate for the evaluated parameters. This high standard highlights effective water resource management, ensuring the sustainability of this vital drinking water source while maintaining ecological balance.

Despite its ecological significance, Jiangxin Island remains vulnerable to flooding because of its unique topography. Historical records indicate two major incidents of riverbank collapse in 2012 and 2020, where floodwaters intruded into the island. However, the swift and coordinated response of government agencies successfully mitigated potential damage, safeguarding both the local population and infrastructure. These incidents highlighted the need for the continuous monitoring and reinforcement of flood prevention measures to enhance the island’s resilience to extreme weather events.

Moreover, Jiangxin Island makes a considerable contribution to biodiversity conservation as part of the Zhenjiang Baiji Dolphin Sanctuary. With low industrial activity, clean water, and abundant fish resources, the sanctuary provides a favorable habitat for the endangered Yangtze River dolphin. A survey by the Freshwater Fisheries Research Center in November 2020 recorded 12 dolphin groups (38 individuals), including mother-calf pairs. Additional sightings in November 2022 confirmed active social behavior, reinforcing the effectiveness of conservation measures and the ecological value of the area.

In terms of vegetation diversity, a survey conducted in Juzhouli, an artificially modified section of the island, revealed low species diversity and an unstable plant community structure (Table 15). Across 10 sampled plots, a total of 78 individual plants belonging to 15 species were recorded. The Shannon–Wiener Diversity Index (H′) of the combined sample was 2.16, while the Pielou’s evenness index (J′) was 0.40, indicating uneven species distribution and limited biodiversity. The predominance of a few species suggests that anthropogenic interventions may have disrupted the dynamics of natural vegetation dynamics. These findings highlight the need to develop ecological restoration strategies for improving plant diversity and promoting a more stable ecosystem.

**Table 15:** Plant diversity indices for the sampled plots.

	Plot 1	Plot 2	Plot 3	Plot 4	Plot 5	Plot 6	Plot 7	Plot 8	Plot 9	Plot 10	Combined sample
Individuals	6	7	9	9	5	18	6	7	2	9	78
Species	2	4	3	5	1	1	2	2	2	4	15
Simpson_1-D	0.60	0.81	0.64	0.83	0	0	0.60	0.48	1	0.69	0.81
Shannon_H	0.78	1.49	1.05	1.69	0	0	0.78	0.67	0.94	1.32	2.16
Pielou’s evenness (J')	0.36	0.69	0.49	0.78	0	0	0.36	0.31	0.44	0.61	0.40

**Fig. 3:**
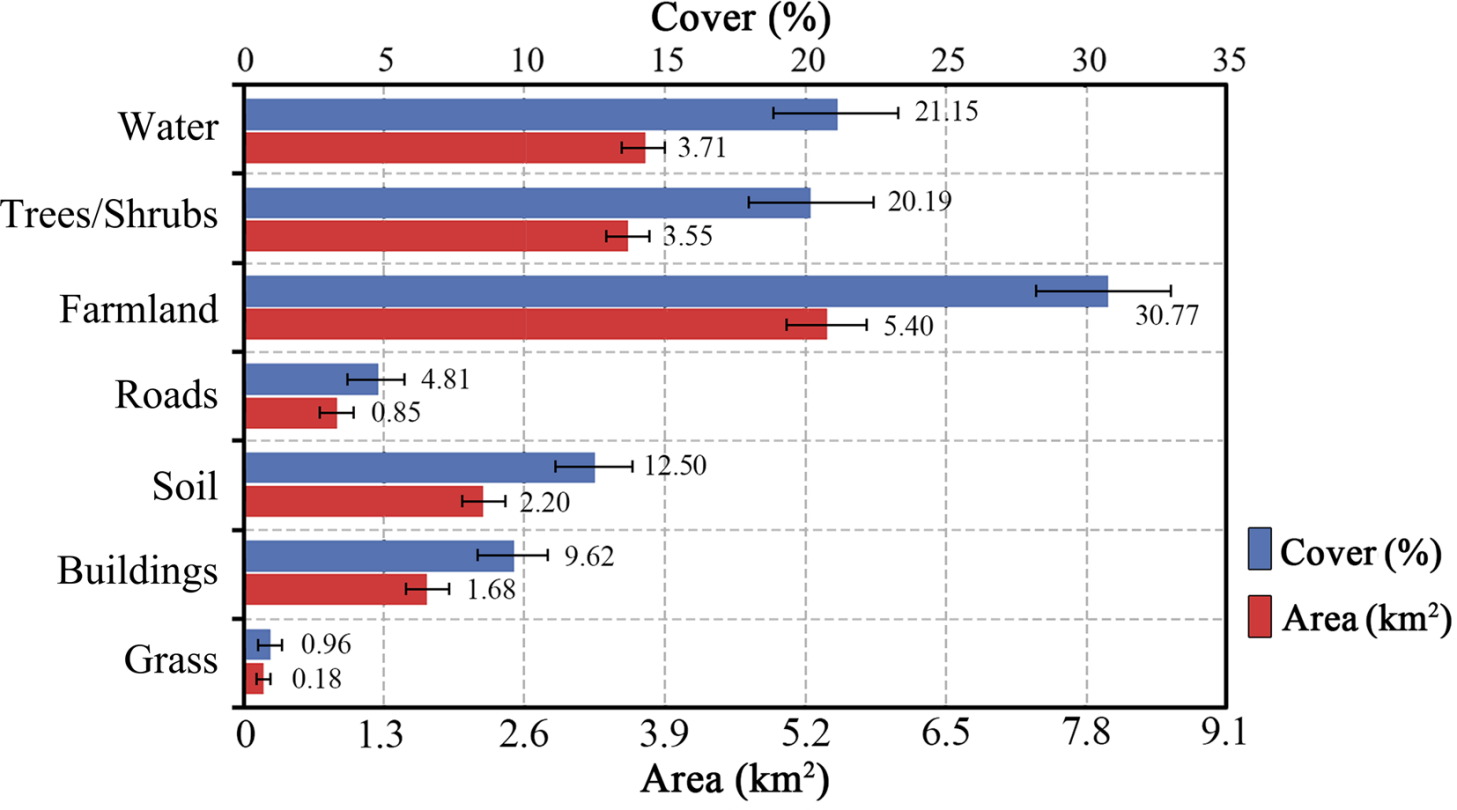
Proportion and area of land cover types.

The land cover composition of the island further reflected a balance between natural and human-modified landscapes. A random sampling of 101 points using i-Tree Canopy analysis yielded the following results regarding the land cover composition: the grassland, buildings, soil, roads, farmland, trees/ shrubs, and water cover percentages were 0.96% ± 0.96%, 9.62 ± 2.890%, 12.50 ± 3.24%, 4.81 ± 2.15%, 30.77 ± 4.53%, 20.19 ± 3.94%, and 21.15 ± 4.00%, respectively (Fig. 3). Additionally, based on the data obtained from the Jiangxin Island Ecological Agricultural Park Management Committee (response of September 7), the island has approximately 5,300 acres of forest, including orchards and forestry land. Moreover, cultivated land covers 11,300 acres, with 6,165 acres officially certified and 6,365 acres having confirmed land rights. The substantial green space coverage and a relatively high proportion of farmland highlighted the dual role of the island as both an ecological preserve and an agricultural resource.

**Fig. 4:**
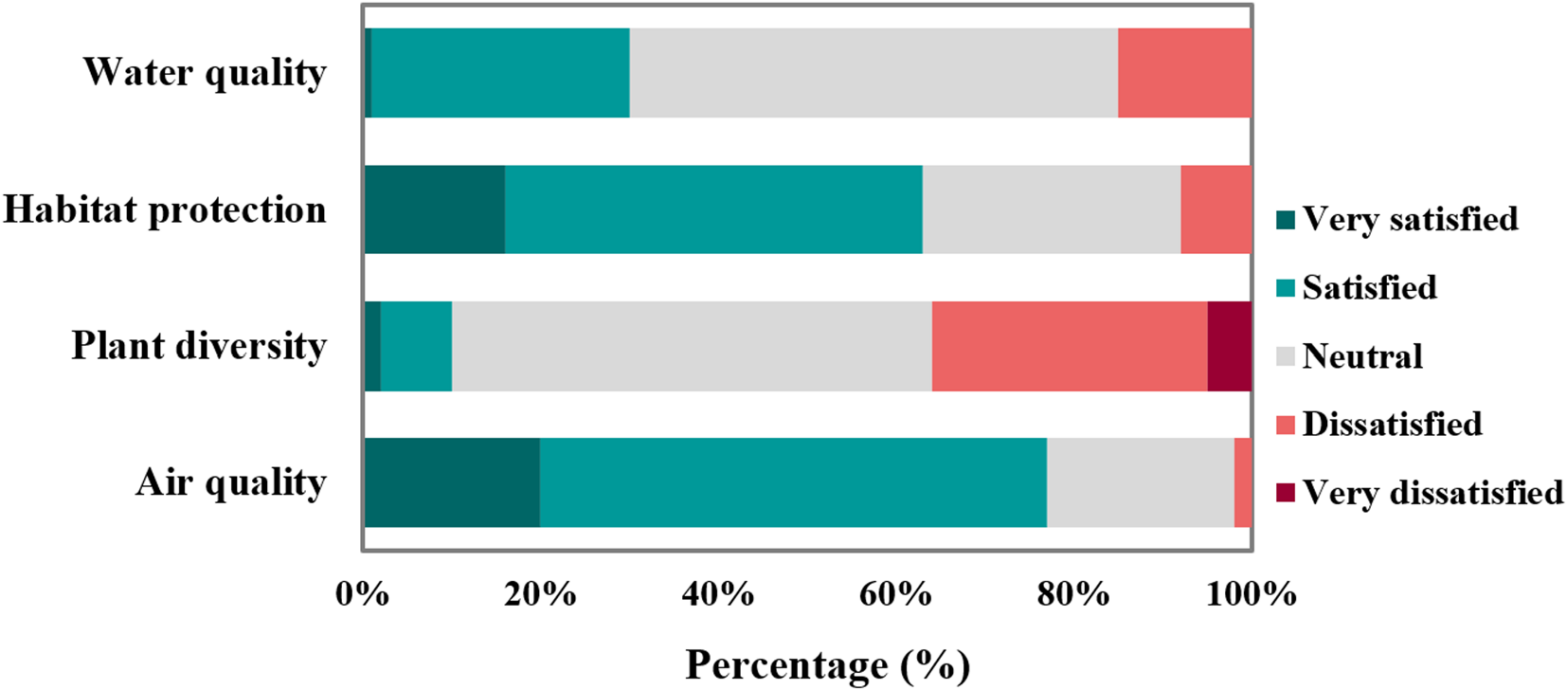
Visitor evaluation of environmental performance.

Our field investigation revealed that tourists frequently engaged in water-related activities such as fishing and boating, making water quality a key factor in their experience. The survey results showed that although 55% rated the water quality as Neutral, 29% were Satisfied, and 15% were Dissatisfied, with a composite score of 4.50, suggesting that although the water quality meets environmental standards, public perception remains mixed (Fig. 3). The satisfaction of visitors with habitat protection followed a similar trend, with 47% satisfied, 29% rating it as Neutral, and 16% Very satisfied, whereas no respondents expressed Very dissatisfied (Fig. 4). Regarding plant diversity, 8% were satisfied,  54% found it Neutral, and 31% expressed Dissatisfaction, indicating that although the vegetation is appreciated, further improvement is needed. Air quality, influenced by nearby industrial facilities, received a largely positive assessment. 57% of respondents were Satisfied, 20% Very satisfied, and only 2% Dissatisfied, resulting in a composite score of 3.80. Overall, although the environmental conditions of Jiangxin Island, particularly air quality and habitat protection, were well-received, the perceptions of water quality and plant diversity highlighted areas for further enhancement.

#### Social performance evaluation

**Fig. 5:**
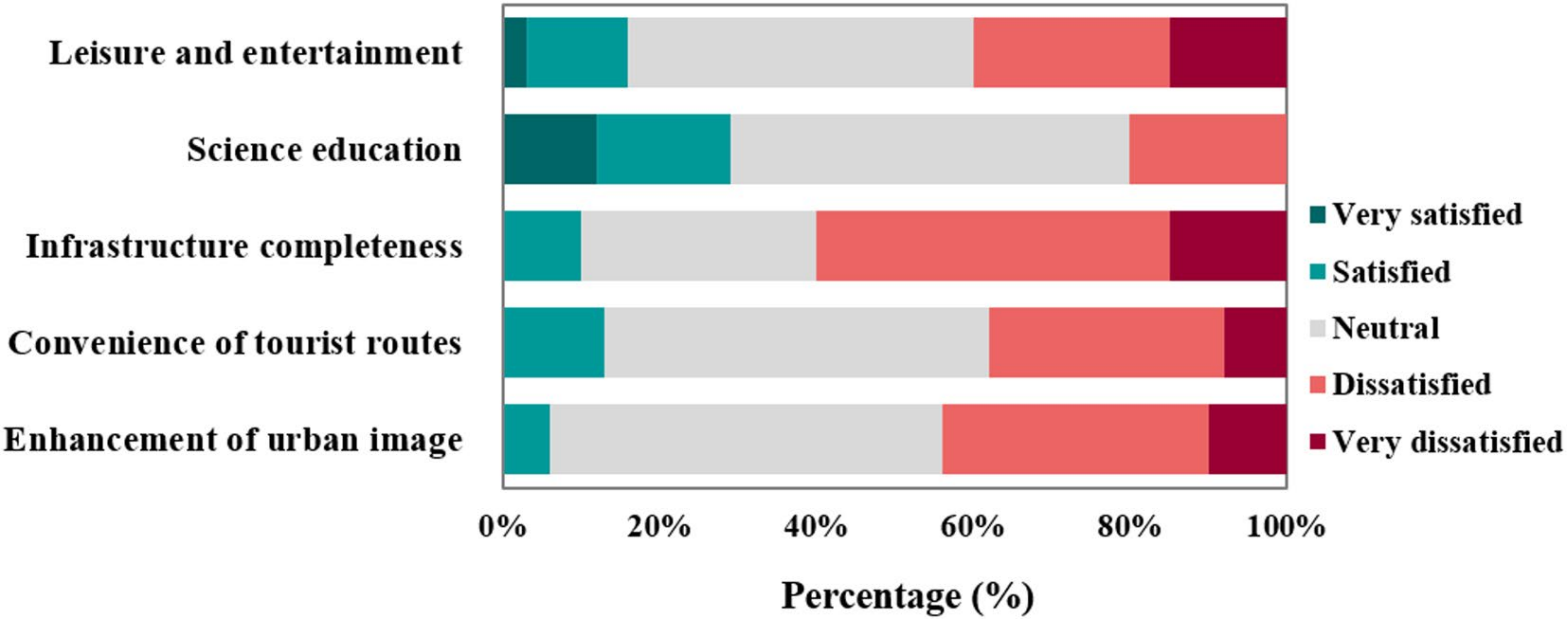
Visitor evaluation of social performance.

The evaluation of leisure and entertainment value in the rural tourism area of Jiangxin Island reveals a generally low level of satisfaction (Fig. 5). According to the survey, 40% of respondents expressed Dissatisfaction (15% Very dissatisfied and 25% Dissatisfied), 44% gave an Neutral rating, and only 16% reported Satisfaction. The composite score of 2.64 highlights the inadequacy of current facilities, suggesting the need for improvements to enrich the visitor experience. Regarding educational value, 17% expressing Satisfaction, 12% being Very satisfied, 20% indicating Dissatisfaction, and 51% rating it as Neutral. Although the initial composite score was 3.21, a relatively more detailed follow-up assessment revealed much lower satisfaction, underscoring the need for better-designed educational experiences. Infrastructure completeness performed poorly, with 60% (15% Very dissatisfied and 45% Dissatisfied) expressing dissatisfaction and a score of only 2.35.

The evaluation of tourism route convenience also indicates underperformance. Although 49% of respondents rated accessibility as Neutral, 30% were Dissatisfied, and only 13% expressed as Satisfied, resulting in a composite score of 2.65. These findings suggest that issues related to wayfinding, signage, or physical access may be limiting overall mobility. Furthermore, the contribution of the rural tourism area to enhancing the urban image was perceived as weak. Half of the respondents rated this as Neutral, 34% as Dissatisfied, 10% as Very dissatisfied, and only 6% as Satisfied. The low score of 2.52 highlighted the need for relatively more strategic branding, improved visual identity, and stronger integration with the city’s tourism positioning.

#### Economic performance evaluation

Interviews with local stakeholders on Jiangxin Island revealed that 412 individual businesses operated on the island, employing approximately 670 people. These businesses primarily provided services such as catering, accommodation, and entertainment for tourists. Before the coronavirus disease (COVID-19) pandemic in 2020, tourism was a major driver of local employment, with nearly every household engaged in tourism-related activities, effectively addressing employment- and income-related issues. The industry significantly contributed to regional economic vitality by creating jobs and retaining residents. However, the pandemic led to a significant decline in tourist numbers, severely impacting the tourism sector and causing a notable outflow of workers.

**Fig. 6:**
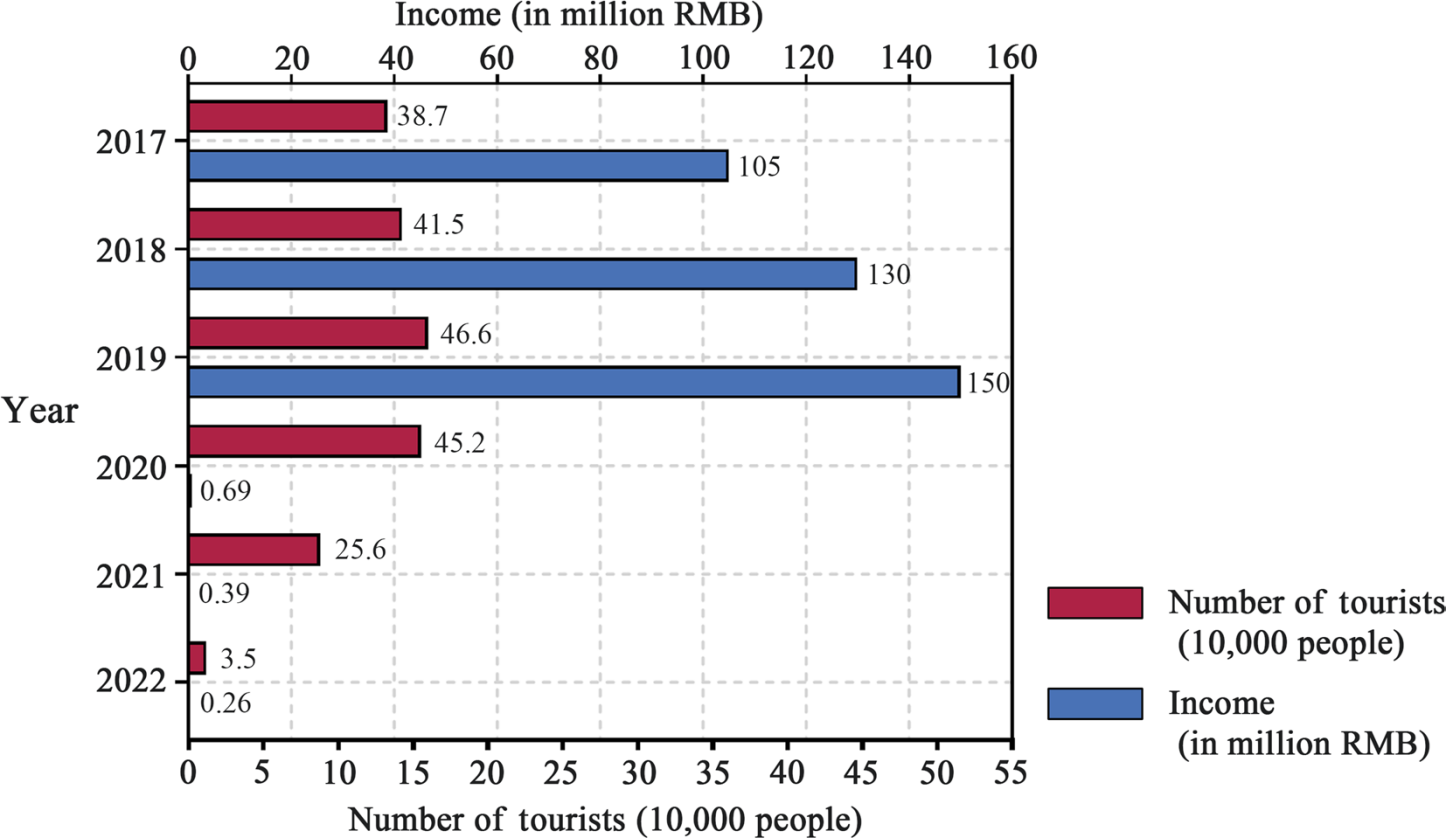
Tourism numbers and tourism revenue on Jiangxin Island.

Regarding tourism consumption, data from the Zhenjiang Municipal People’s Government (released on September 7, 2020) highlighted key statistics on rural tourism employment, income, visitor numbers, and spending trends (Fig. 6). In the first half of 2022, the Jiangxin Island scenic area and rural tourism zone were closed from March 15 to May 23 because of COVID-19. During this period, the total number of visitors amounted to 35,000, generating a tourism revenue of RMB 260,000. Although tourism revenue declined during the pandemic period, the steady upward trend observed before 2019 suggests that Jiangxin Island had been on a path of gradual tourism growth, indicating potential for continued development under stable conditions.

#### Aesthetic performance evaluation

**Fig. 7:**
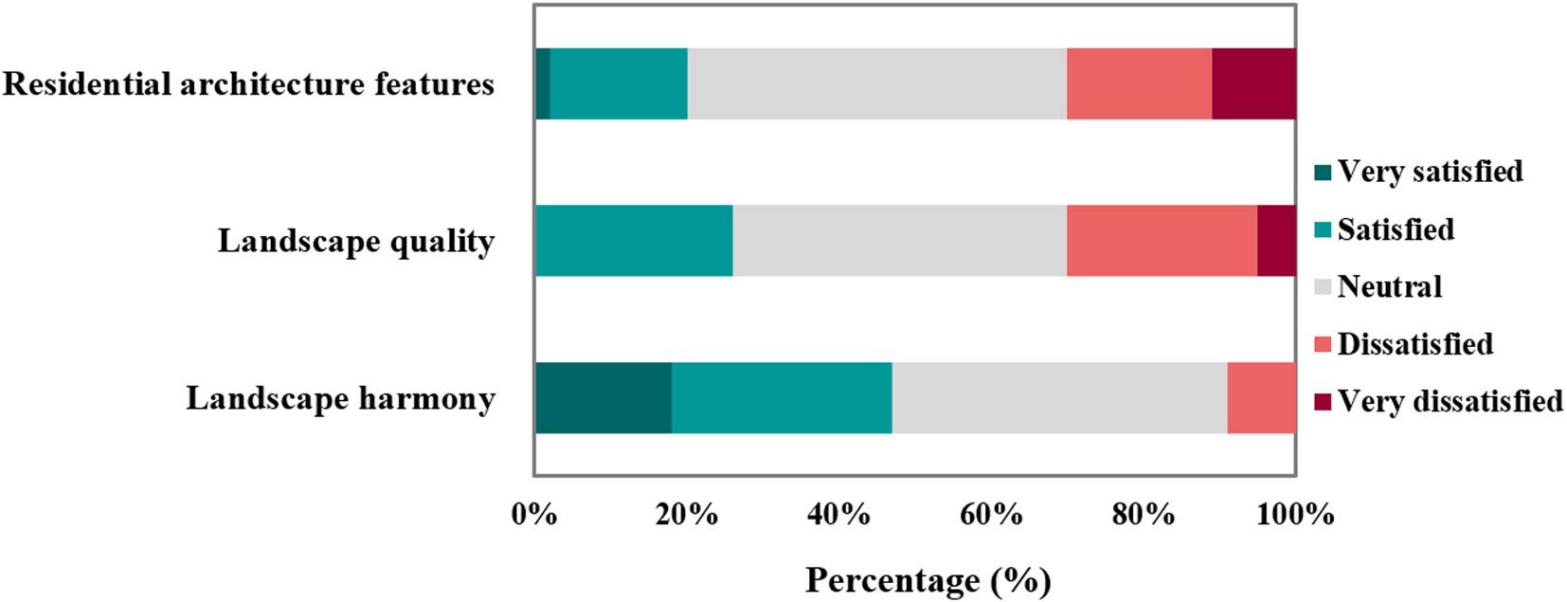
Visitor evaluation of aesthetic performance.

The evaluation of aesthetic performance in the Jiangxin Island rural tourism area reveals varied levels of visitor satisfaction across different components. Regarding residential architectural features, although 50% of respondents rated them as Neutral, 30% expressed dissatisfaction (11% Very dissatisfied and 19% Dissatisfied), whereas only 18% were Satisfied and 2% were Very satisfied. This distribution revealed a clear need to enhance the architectural feature and visual coherence of buildings, ensuring that the local identity is better reflected. The resulting index score of 2.82 reflects this overall moderate visitor perception, as illustrated in Fig. 7.

Similarly, the evaluation of landscape quality indicates a need for improvement. Although 44% of respondents rated it as Neutral and 26% expressed satisfaction, 30% reported dissatisfaction (5% Very dissatisfied and 25% Dissatisfied). The overall index score of 2.91 again indicates a mid-level performance that lacks strong approval. These results suggest that the scenic and design elements may not fully meet visitors’ expectations for visual richness and diversity.

In contrast, landscape harmony was perceived relatively more positively. A combined 47% of respondents indicated satisfaction (18% Very satisfied and 29% Satisfied) compared with only 9% expressing dissatisfaction. The index score of 3.56 suggests a generally favorable view of how natural and built elements are integrated, though further refinements could enhance landscape harmony.

### LPE results for the tourism-oriented rural area of Jiangxin Island

**Fig. 8:**
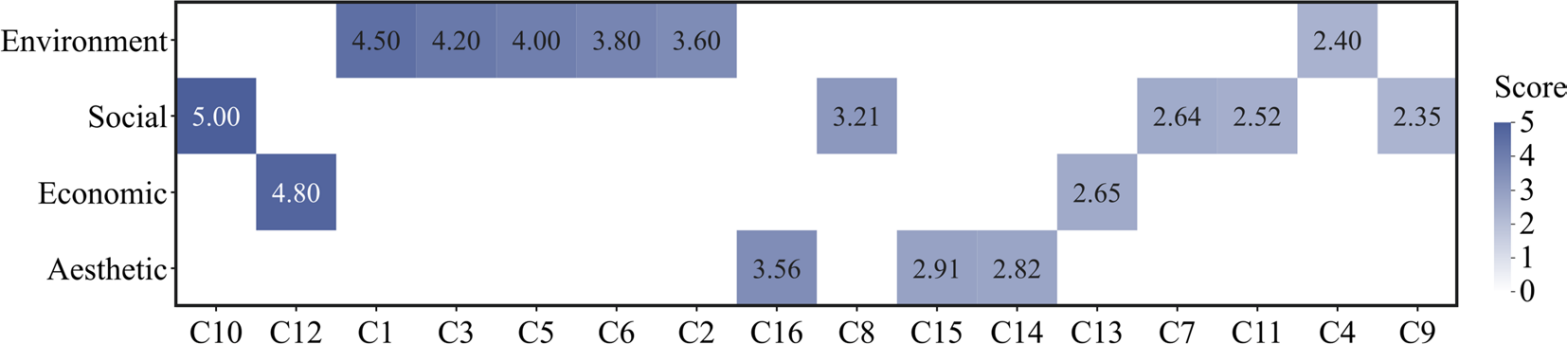
Heatmap of the composite LPE score for Jiangxin Island.

Based on a comprehensive analysis of the Jiangxin Island—encompassing environmental, economic, social, and aesthetic aspects—along with the evaluation criteria for various indicators, an LPE was conducted. The analysis incorporated data from visitor surveys, expert questionnaires, and government-disclosed information at different administrative levels. The integrated index evaluation model was applied to quantify the landscape performance, with the results summarized in Table 16 and Fig. 8.

**Table 16:** Composite score of the LPE for Jiangxin Island.

Goal layer	Criteria layer	Indicator layer	Weight value	Score
LPE framework for tourism-oriented rural area of Jiangxin Island	Environment performance (B1)	Water quality (C1)	0.06	4.50
		Flood control (C2)	0.04	3.60
		Habitat protection (C3)	0.06	4.20
		Plant diversity (C4)	0.05	2.40
		Green space ratio (C5)	0.07	4.00
		Air quality (C6)	0.05	3.80
	Social performance (B2)	Leisure and entertainment (C7)	0.08	2.64
		Science education (C8)	0.07	3.21
		Infrastructure completeness (C9)	0.06	2.35
		Convenience of tourist routes (C10)	0.05	2.65
		Enhancement of urban image (C11)	0.22	2.52
	Economic performance (B3)	Job creation (C12)	0.06	4.80
		Tourism consumption (C13)	0.18	5.00
	Aesthetic performance (B4)	Residential architectural features (C14)	0.06	2.82
		Landscape quality (C15)	0.06	2.91
		Landscape harmony (C16)	0.03	3.56

Based on the scoring criteria of the integrated index method, a higher score indicates stronger performance in various dimensions of the rural tourism of Jiangxin Island. The results highlight notable achievements in environmental protection, sustainable development, and economic contributions, particularly in job creation and tourism revenue. However, relatively low scores in aspects such as plant diversity, infrastructure development, and urban image enhancement revealed areas in need of improvement. The comprehensive index for LPE was calculated by weighting the scores of each indicator, with the final results presented in Table 17.

**Table 17:** LPE composite score for Jiangxin Island.

Goal layer	Score	Criteria layer	Comprehensive weights	Score
LPE framework for tourism-oriented rural area of Jiangxin Island	3.66	Environment performance (B1)	0.34	3.81
		Social performance (B2)	0.28	2.72
		Economic performance (B3)	0.24	4.95
		Aesthetic performance (B4)	0.14	3.00

The overall score of 3.66 indicates that although Jiangxin Island demonstrates a moderate-to-good level of landscape performance, improvements in social and aesthetic aspects are still needed to enhance the overall tourism experience.

## Discussion

### Establishing a LPE framework for tourism-oriented rural areas

In this study, we established a comprehensive LPE framework for tourism-oriented rural areas and applied it to Jiangxin Island in Zhenjiang, Jiangsu Province, China, as a case study. The overall score for Jiangxin Island was 3.66, classifying it as Good level based on the established evaluation scale (Table 13). This result indicates that the area performs well across environmental, economic, social, and aesthetic dimensions, demonstrating satisfactory ecological protection, an improvement in residents’ income, positive visitor feedback, and an overall high landscape quality. However, a comparison of the different criteria layers reveals significant variations in their performance. The environmental performance criteria exhibited the highest weight (0.34) and a score of 3.81, ranking second among all indicators, suggesting that Jiangxin Island demonstrates relatively strong environmental protection performance, particularly in terms of water quality, air quality, and habitat conservation. These findings align with those of Germaine et al.^40^, who reported that forested and agricultural landscapes serve as key attractors of ecotourism and contribute to sustainable development outcomes. However, these findings are inconsistent with those of Shang and Xu^41^, who highlighted the pollution impact of ecotourism on scenic spots. Similarly, a case study of Hainan Island highlighted the spatial coupling of rural tourism and ecological agriculture, emphasizing the importance of coordinated development to ensure long-term sustainability^42^. The study reported that although some regions exhibit strong ecological performance owing to tourism-driven conservation efforts, disparities between northern and southern areas remain evident, suggesting that regional policies must account for spatial variations in tourism–agriculture interactions to optimize sustainability^42^. The results obtained in our study suggest that Jiangxin Island maintains a balanced environmental performance, likely because of local conservation policies and controlled tourism development.

The economic performance dimension received the highest score (4.95) with a weight of 0.24, suggesting a strong role of tourism in supporting local employment and economic activity, as reflected in expert evaluations. This finding is consistent with Paresishvili^43^, who emphasized rural tourism as a key driver of local economic growth. Furthermore, it aligns with Gao^44^, who found that tourism development in traditional villages significantly enhances local economies. A similar trend was observed in Guangxi’s Chengyang Bazhai, where Wang et al. ^45^ reported that rapid tourism expansion initially led to a decline in the ecological-production-living space (EPLS)-carrying capacity in the region, despite increasing economic benefits. However, after 2015, EPLS carrying capacity stabilized, aligning with a more sustainable economic development trajectory, highlighting the need to balance tourism growth with environmental sustainability for long-term economic resilience^45^. Unlike studies suggesting that rural tourism brings short-term economic benefits, Jiangxin Island’s sustained high economic performance may be attributed to diversified revenue sources, including accommodations, recreational activities, and government incentives for tourism development^46^. In contrast, social performance received the lowest score (2.72) despite its relatively high weight (0.28), suggesting deficiencies in aspects such as visitor convenience, infrastructure development, and cultural engagement. This result contrasts with Kim^47^, who found that well-integrated rural tourism often enhances community well-being. A likely explanation for this discrepancy is that the tourism planning of Jiangxin Island prioritizes economic and environmental goals over community-oriented development, resulting in challenges in infrastructure accessibility and local engagement. Future improvements could focus on enhancing tourism facilities, improving transportation accessibility, and increasing community participation to ensure improved integration of social benefits into the tourism model. The aesthetic performance dimension, with the lowest weight (0.14) and a moderate score (3.00), suggests that while landscape quality and residential architectural features contribute to overall satisfaction, they play a relatively minor role in influencing the comprehensive landscape performance. These findings align with those of Jiang et al.^48^, who noted that visual quality significantly affects tourists’ preferences but is often secondary to functional aspects, such as “facility” and “maintenance.” However, unlike Howley^21^, who found that public preferences are strongest for water-related landscapes followed by cultural landscapes, this study suggests that aesthetic preferences in tourism-oriented rural areas may be more context-dependent. Given the diversity of preferences identified, a standardized approach to landscape planning may not fully capture the varied expectations of different visitor groups, highlighting the need for tailored strategies that balance aesthetic appeal with functional considerations.

Overall, the findings demonstrate that the proposed framework successfully captures the multifaceted nature of landscape performance in tourism-oriented rural areas. The differentiated results across environmental, economic, social, and aesthetic dimensions validate the relevance of the indicator system and weighting scheme established in the conceptual framework. The alignment between expert judgment and field-based perception data supports the robustness of the evaluation approach. However, the relatively low scores in social and aesthetic aspects also indicate areas where the framework may require further refinement to better account for experiential and cultural dimensions. While Jiangxin Island performs well in economic and environmental terms, future strategies should focus on improving infrastructure, cultural engagement, and visual quality to enhance visitor satisfaction and long-term sustainability. Although this study centers on Jiangxin Island, the proposed framework is adaptable to other rural tourism contexts and offers practical value for broader landscape planning and policy development.

### Implication of LPE framework for Jiangxin Island

A detailed analysis of the evaluation results reveals both strengths and deficiencies in the Jiangxin Island, offering direct validation of the multidimensional LPE framework proposed in this study. Water quality received a high score, reflecting effective conservation efforts and alignment with the environmental protection goals embedded in the framework. However, the relatively low score for flood control capacity highlights the need for enhanced infrastructure and adaptive strategies to mitigate climate-related risks, reinforcing the framework’s emphasis on resilience. Among environmental indicators, plant diversity ranked low, suggesting deficiencies in species selection and spatial distribution. Inappropriate planting not only obstructs scenic views but also contributes to biodiversity loss and soil degradation, stressing the need for a strategic design approach grounded in ecological function and aesthetic perception.

In terms of social performance, infrastructure satisfaction ranked the lowest, indicating that current facilities do not meet visitor expectations. Leisure and entertainment value, along with tourism accessibility, also performed poorly, limiting engagement and suggesting gaps in addressing the social dimension outlined in the LPE model. Furthermore, the low score for urban image enhancement reflects the underdeveloped regional identity and branding capacity of this area. These findings suggest that although the framework captures comprehensive performance, actual planning and implementation in Jiangxin Island remain unbalanced across dimensions.

By contrast, economic indicators performed strongly, with job creation and tourism consumption scoring high, which validates the economic evaluation logic within the framework and demonstrates that rural tourism can effectively drive local economic development. However, dependence on traditional sectors—such as agriculture and basic lodging—signals the need to diversify revenue streams through cultural experiences, ecotourism, and local creative industries for long-term sustainability. Aesthetic performance, particularly residential architectural features, was also poorly rated. Many farmstays and homestays lack distinct regional characteristics, revealing an implementation gap in capturing cultural identity, a key component in the aesthetic dimension of the framework. The low scores in social and aesthetic dimensions may reflect limited cultural expression, a weak sense of place, and a lack of emotionally engaging spaces. Prior studies have highlighted that local identity, multisensory experience, and community-based design are key to enhancing visitor satisfaction and landscape perception^21,22^. Strengthening these elements can help improve the experiential and cultural value of the landscape.

To enhance the overall landscape performance of Jiangxin Island and further operationalize the LPE framework, targeted interventions should be implemented across key dimensions: (1) Enhancing environmental resilience through flood mitigation and optimized plant selection, including nature-based solutions such as wetland restoration; (2) Improving infrastructure and services by upgrading transportation, recreation, and educational amenities, with support from smart tourism technologies; (3) Diversifying economic opportunities via high-value tourism products like cultural heritage tours and local craft markets; and (4) Strengthening aesthetic identity by embedding local cultural elements in architecture, refining vegetation strategies, and using sustainable building materials.

### Limitations and perspectives

This study developed an LPE framework for tourism-oriented rural areas and applied it to Jiangxin Island in Zhenjiang. While the framework integrates environmental, economic, social, and aesthetic dimensions, several limitations remain, which also point to key areas for future research: (1) Limited data scope: This study pays relatively limited attention to socio-cultural factors, potentially overlooking critical human–land interactions. Incorporating multi-source data, such as local culture, community participation, and tourist experiences, would help enhance the comprehensiveness of the framework; (2) Methodological constraints: Some indicators are static, which limits their ability to reflect long-term changes in landscape performance. Future studies should incorporate dynamic models, real-time monitoring, and long-term tracking to assess seasonal and socio-economic shifts. Additionally, while AHP offers transparency and interpretability, its reliance on expert judgment introduces subjectivity. Machine learning methods such as random forest or clustering algorithms could be considered complementary or alternative approaches for indicator weighting and pattern discovery in complex datasets; (3) Limited generalizability: Although based on a single case study, the framework’s modular structure allows for adaptation to other regions by adjusting indicator sets and weights. Future applications can incorporate locally relevant factors—such as cultural heritage, land-use practices, or community values—to enhance contextual relevance and support broader implementation; (4) Insufficient stakeholder engagement: Although stakeholder perspectives were considered, broader public participation is needed to ensure the framework reflects diverse needs. Future research should establish structured dialogue mechanisms with local communities, policymakers, and tourists, integrating participatory approaches into landscape evaluation and planning. Such participatory methods would enhance the accuracy, adaptability, and relevance of the framework, supporting the sustainable and resilient development of tourism-oriented rural destinations; and (5) Expert panel size in AHP: The AHP-based analysis drew on inputs from 12 experts. While this is a common practice in expert-driven AHP-based studies^49^, the limited panel size may constrain the diversity of perspectives. Future research could address this by expanding the panel, applying fuzzy AHP, or incorporating participatory methods such as stakeholder interviews. Additionally, future research should test the framework’s transferability to other rural regions and clarify its practical relevance for policymakers seeking to align rural tourism with sustainable agricultural management.

## Conclusion

In this study, we developed an LPE framework for tourism-oriented rural areas by integrating performance theory, rural revitalization strategies, and sustainable development principles. Employing AHP-based analyses and the composite index method, we established a multidimensional evaluation system and applied it to the tourism-oriented rural area of Jiangxin Island. The results provide both quantitative and qualitative insights into the environmental, social, economic, and aesthetic performance of the site, which will be useful for making strategic recommendations for landscape improvement and tourism management. The main conclusions are as follows:

1. Development of a comprehensive LPE framework for tourism-oriented rural areas

A structured and adaptable evaluation model was constructed by synthesizing existing theories and tools from landscape performance, rural tourism, and sustainability research. The 16 indicators grouped into four core dimensions—environmental, social, economic, and aesthetic—reflect the holistic and integrated nature of rural landscape systems. This framework advances theoretical development by bridging sectoral gaps and formalizing an interdisciplinary structure for assessing rural tourism landscapes. Nevertheless, it remains partly limited by the reliance on expert-based weighting and context-specific indicator calibration, which may affect generalizability in broader or culturally distinct regions.

(2) Empirical validation through the Jiangxin Island case study

The case study demonstrated the applicability, practicality, and diagnostic value of the framework. The use of mixed methods, including surveys, remote sensing, and stakeholder interviews, helped ensure the robustness of data. Jiangxin Island was rated as performing Good level overall, with notable strengths in economic and environmental aspects, yet apparent deficiencies in social infrastructure and aesthetic identity. These findings validate the framework’s capacity to detect dimensional imbalances and support data-informed decision-making. However, the evaluation also revealed challenges in cross-dimensional integration and highlighted the need for continuous updating to reflect evolving local conditions.

(3) Contributions and policy implications

The results obtained in this study highlight the interdependence of environmental, economic, social, and aesthetic factors in shaping sustainable rural tourism, emphasizing the need for a planning paradigm that balances ecological resilience, economic diversification, and cultural expression. The framework offers theoretical contributions by consolidating performance-based and value-driven assessment principles, while also providing actionable guidance for planners and policymakers. Future improvements could explore dynamic weighting systems, participatory evaluation mechanisms, and spatial–temporal scalability to enhance the applicability of this framework. Through this integrative approach, the study contributes to expanding methodological pathways and practical insights for evaluating and improving rural landscape sustainability in tourism-oriented regions. It also offers a reference for linking agricultural land use with rural tourism to support sustainable development.

## Supplementary Information

Below is the link to the electronic supplementary material.

Supplementary Material 1

## Data Availability

All data generated or analyzed during this study is provided within the manuscript or supplementary information files.
