# Supplementary material for: An integrated framework for evaluating landscape performance in tourism-oriented rural areas
Source: Sci Rep. 2025 Dec 8;15:45794. doi: 10.1038/s41598-025-28784-y (PMC12756229; doi:10.1038/s41598-025-28784-y)
Supplement: Supplementary file 1 — Supplementary Material 1 [file 41598_2025_28784_MOESM1_ESM.docx]

**Appendix**

**Table A1 Jiangxin Island tourism resources**

| **Main Category** | **Subcategory** | **Basic Type** | **Brief Description** |
| --- | --- | --- | --- |
| A. Geomorphological Landscapes | AA. Natural Landscape Complex | AAD. Shoal-type Landscape | Overall or individual landscapes on gentle shoals, suitable for sightseeing and tourism |
| B. Water Landscapes | BA. River Systems | BAA. Recreational River Sections | River segments suitable for sightseeing and tourism |
|  | BB. Lakes and Marshes | BBA. Recreational Lake Areas | Lake water areas designated for sightseeing and tourism |
|  |  | BBC. Wetlands | Natural or artificial marshlands with still or flowing shallow water bodies |
| C. Biological Landscapes | CA. Vegetation Landscapes | CAA. Forests | Yangtze River Shelterbelt |
|  |  | CAC. Grasslands | Jujingli Tourism Area |
|  | CB. Wildlife Habitats | CBA. Aquatic Wildlife Habitats | Provincial Finless Porpoise Nature Reserve |
| E. Architectural and Facility Landscapes | EA. Comprehensive Human Landscape Complex | EAF. Wellness, Entertainment, and Leisure Resorts | Jujingli, Baizhiwei Farm, Agritainment |
|  |  | EAC. Educational and Research Experimental Sites | Herb Museum |
|  | EB. Functional Buildings and Core Facilities | EBB. Distinctive Houses | Stargazing House, Tent Hotel |
|  |  | EBE. Bridges | Lotus Viewing Bridge |
|  |  | EBF. Channels and Canal Sections |  |
|  |  | EBG. Dikes and Embankments | Flood Control Dike |
|  |  | EBH. Ports, Ferry Terminals, and Docks | Jiangxin Ferry Terminal |
|  |  | EBK. Scenic Farmlands | Green Corridor |
|  | EC. Scenic and Small-scale Architectural Structures | ECC. Terraces, Towers, and Pavilions | Zhiqing Cultural Wall |
|  |  | ECJ. Scenic Trails and Pathways | Jujingli Tourism Area |
| F. Historical Sites | FA. Tangible Cultural Heritage | FAA. Architectural Relics |  |
| G. Tourism Shopping | GA. Agricultural Products | GAA. Crop-based Products and Goods | "Tanbayang" pollution-free vegetables, seasonal fruits, persimmon orchards, ginkgo groves |
|  |  | GAE. Aquaculture Products and Goods | National Geographical Indication “Zhenjiang River Crab” breeding base |

**Table A2 Expert consultation questionnaire for the LPE of Jiangxin Island**

Dear Experts,

I am very grateful that you have taken valuable time from your busy schedule to assist in this study on developing a landscape performance evaluation (LPE) framework for tourism-oriented rural areas. We intend to use the analytic hierarchy process (AHP) to determine the weights of the LPE framework for the rural tourism area of Jiangxin Island.

Specifically, the evaluation framework is presented in Table B1: Comprehensive LPE framework for the Jiangxin Island Rural Tourism Area, and Table B2: Judgment Matrix Scale provides the paired comparison scale used for scoring. Based on these tables, please score the criteria layer and the corresponding indicator layer in pairs.

Thank you very much for your support!

**Table B1** Comprehensive LPE framework for tourism-oriented rural area of Jiangxin Island

| Goal Layer | Criteria Layer | Judgment Matrix (Pairwise) | Indicator Layer | Judgment Matrix (Pairwise) | Description |
| --- | --- | --- | --- | --- | --- |
| LPE framework for tourism-oriented rural area of Jiangxin Island | Environmental Performance (B1) | B1 vs B2, B1 vs B3, B1 vs B4 | Water Quality (C1) | C1 vs C2, C1 vs C3, ... | Water body environmental quality |
|  |  |  | Flood Control (C2) | C2 vs C3, C2 vs C4, ... | Flood control level |
|  |  |  | Habitat Protection (C3) | C3 vs C4, C3 vs C5, ... | Habitat protection conditions within the site |
|  |  |  | Plant Diversity (C4) | C4 vs C5, C4 vs C6, ... | Plant species within the site |
|  |  | B2 vs B3, B2 vs B4  B3 vs B4 | Green Space Rate (C5) | C5 vs C6, C5 vs C7, ... | Proportion of green area to total land area of Jiangxin Island |
|  |  |  | Air Quality (C6) | C6 vs C7, C6 vs C8, ... | Ambient air quality conditions |
|  | Social Performance (B2) |  | Leisure and Entertainment (C7) | C7 vs C8, C7 vs C9, ... | Attractiveness of leisure and entertainment for tourists on Jiangxin Island |
|  |  |  | Science Education (C8) | C8 vs C9, C8 vs C10, ... | Science- and ecology-related educational facilities and activities |
|  |  |  | Infrastructure Completeness (C9) | C9 vs C10, C9 vs C11, ... | Completeness of basic infrastructure on Jiangxin Island |
|  |  |  | Convenience of Tourist Routes (C10) | C10 vs C11, C10 vs C12, ... | Whether the tourist routes are reasonable and have high accessibility |
|  |  |  | Enhancement of Urban Image (C11) | C11 vs C12, C11 vs C13, ... | Role in enhancing the local urban image |
|  | Economic Performance (B3) |  | Job Creation (C12) | C12 vs C13, C12 vs C14, ... | Provision of employment opportunities for residents |
|  |  |  | Tourism Consumption (C13) | C13 vs C14, C13 vs C15, ... | Per capita tourism consumption of the project |
|  | Aesthetic Performance (B4) |  | Residential Architectural Features (C14) | C14 vs C15, C14 vs C16, ... | Architectural style and characteristics within the site |
|  |  |  | Landscape Quality (C15) | C15 vs C16 | Aesthetic degree of landscape creation |
|  |  |  | Landscape Harmony (C16) | — | Degree of harmony between landscape facilities and the surrounding natural environment |

**Explanation of Columns:**

Goal Layer: The top-level category in the evaluation.

Criterion Layer: Lists the criteria that fall under each goal layer.

Indicator Layer: Lists the indicators corresponding to each criterion.

Explanation: Provides a brief description of each indicator.

Judgment Matrix: Contains the pairwise comparison values used in the AHP, representing how indicators are compared with one another.

**Table B2** Judgment matrix scale analysis

| Scale Value | Scale Meaning |
| --- | --- |
| 1 | Indicates that two indicators are equally important |
| 2 | Indicates that the former is weakly more important than the latter |
| 3 | Indicates that the former is moderately more important than the latter |
| 4 | Indicates that the former is strongly more important than the latter |
| 5 | Indicates that the former is extremely more important than the latter |
| Reciprocal | Indicates that the latter is more important than the former (e.g., 1/2, 1/3, 1/4) |

Thank you for your valuable participation in this survey.

**Table A3 Satisfaction survey for tourism-oriented rural area of Jiangxin Island**

| **Five-point Scale** | | | | |
| --- | --- | --- | --- | --- |
| Very Dissatisfied | Dissatisfied | Neutral | Satisfied | Very Satisfied |
| Very Dissatisfied ○1 ○ 2 ○ 3 ○ 4 ○ 5 Very Satisfied | | | | |

* All the following items are single-choice questions.

1. **Your gender**
   - Female
2. **Your age**
   - Under 20
   - 20–44
   - 45–60
   - Above 60
3. **Travel companion type**
   - Solo travel
   - With partner
   - With friends
4. **Where are you from?**
   - Zhenjiang City
   - Within Jiangsu Province
   - Outside Jiangsu Province
5. **How long do you spend visiting the tourism-oriented rural area of Jiangxin Island?**
   - Less than 3 hours
   - 3–6 hours
   - 6–12 hours
   - More than 12 hours
6. **How many times do you visit the tourism-oriented rural area of Jiangxin Island in a year?**
   - 1 time
   - 2–4 times
   - 5–6 times
   - More than 6 times
7. **Are you satisfied with the water quality of the tourism-oriented rural area of Jiangxin Island?**

| Very Dissatisfied | Dissatisfied | Neutral | Satisfied | Very Satisfied |
| --- | --- | --- | --- | --- |
|  |  |  |  |  |

1. **Are you satisfied with the status of the Yangtze finless porpoise habitat protection in the tourism-oriented rural area of Jiangxin Island?**

| Very Dissatisfied | Dissatisfied | Neutral | Satisfied | Very Satisfied |
| --- | --- | --- | --- | --- |
|  |  |  |  |  |

1. **Are you satisfied with the entertainment facilities in the tourism-oriented rural area of Jiangxin Island?**

| Very Dissatisfied | Dissatisfied | Neutral | Satisfied | Very Satisfied |
| --- | --- | --- | --- | --- |
|  |  |  |  |  |

1. **Are you satisfied with the infrastructure of the tourism-oriented rural area of Jiangxin Island?**

| Very Dissatisfied | Dissatisfied | Neutral | Satisfied | Very Satisfied |
| --- | --- | --- | --- | --- |
|  |  |  |  |  |

1. **Are you satisfied with the plant diversity of the tourism-oriented rural area of Jiangxin Island?**

| Very Dissatisfied | Dissatisfied | Neutral | Satisfied | Very Satisfied |
| --- | --- | --- | --- | --- |
|  |  |  |  |  |

1. **Are you satisfied with the agricultural science education and popularization activities in the tourism-oriented rural area of Jiangxin Island?**

| Very Dissatisfied | Dissatisfied | Neutral | Satisfied | Very Satisfied |
| --- | --- | --- | --- | --- |
|  |  |  |  |  |

1. **Are you satisfied with the convenience of the tourism routes in the tourism-oriented rural area of Jiangxin Island?**

| Very Dissatisfied | Dissatisfied | Neutral | Satisfied | Very Satisfied |
| --- | --- | --- | --- | --- |
|  |  |  |  |  |

1. **How satisfied are you with the role of the tourism-oriented rural area of Jiangxin Island in enhancing the image of Zhenjiang City?**

| Very Dissatisfied | Dissatisfied | Neutral | Satisfied | Very Satisfied |
| --- | --- | --- | --- | --- |
|  |  |  |  |  |

1. **Are you satisfied with the fruit and agricultural product experience projects in the tourism-oriented rural area of Jiangxin Island?**

| Very Dissatisfied | Dissatisfied | Neutral | Satisfied | Very Satisfied |
| --- | --- | --- | --- | --- |
|  |  |  |  |  |

1. **Are you satisfied with the overall landscape quality of the tourism-oriented rural area of Jiangxin Island?**

| Very Dissatisfied | Dissatisfied | Neutral | Satisfied | Very Satisfied |
| --- | --- | --- | --- | --- |
|  |  |  |  |  |

1. **Are you satisfied with the residential architectural features in the tourism-oriented rural area of Jiangxin Island?**

| Very Dissatisfied | Dissatisfied | Neutral | Satisfied | Very Satisfied |
| --- | --- | --- | --- | --- |
|  |  |  |  |  |

1. **Are you satisfied with the harmony between the scenic area facilities and the natural environment in the tourism-oriented rural area of Jiangxin Island?**

| Very Dissatisfied | Dissatisfied | Neutral | Satisfied | Very Satisfied |
| --- | --- | --- | --- | --- |
|  |  |  |  |  |

Through your visit, what aspects of the scenic area do you think need improvement, and what are your suggestions for improvement? (Optional)
